# Supplementary material for: Comprehensive Annotation and Functional Exploration of MicroRNAs in Lettuce
Source: Front Plant Sci. 2021 Dec 24;12:781836. doi: 10.3389/fpls.2021.781836 (PMC8739914; doi:10.3389/fpls.2021.781836)
Supplement: Supplementary file 2 [file Data_Sheet_8.ZIP › GSM2230749.plot/Lsa-miRN41_Lsat_1_v5_gn_6_28920.1_4912_TPlot.pdf]

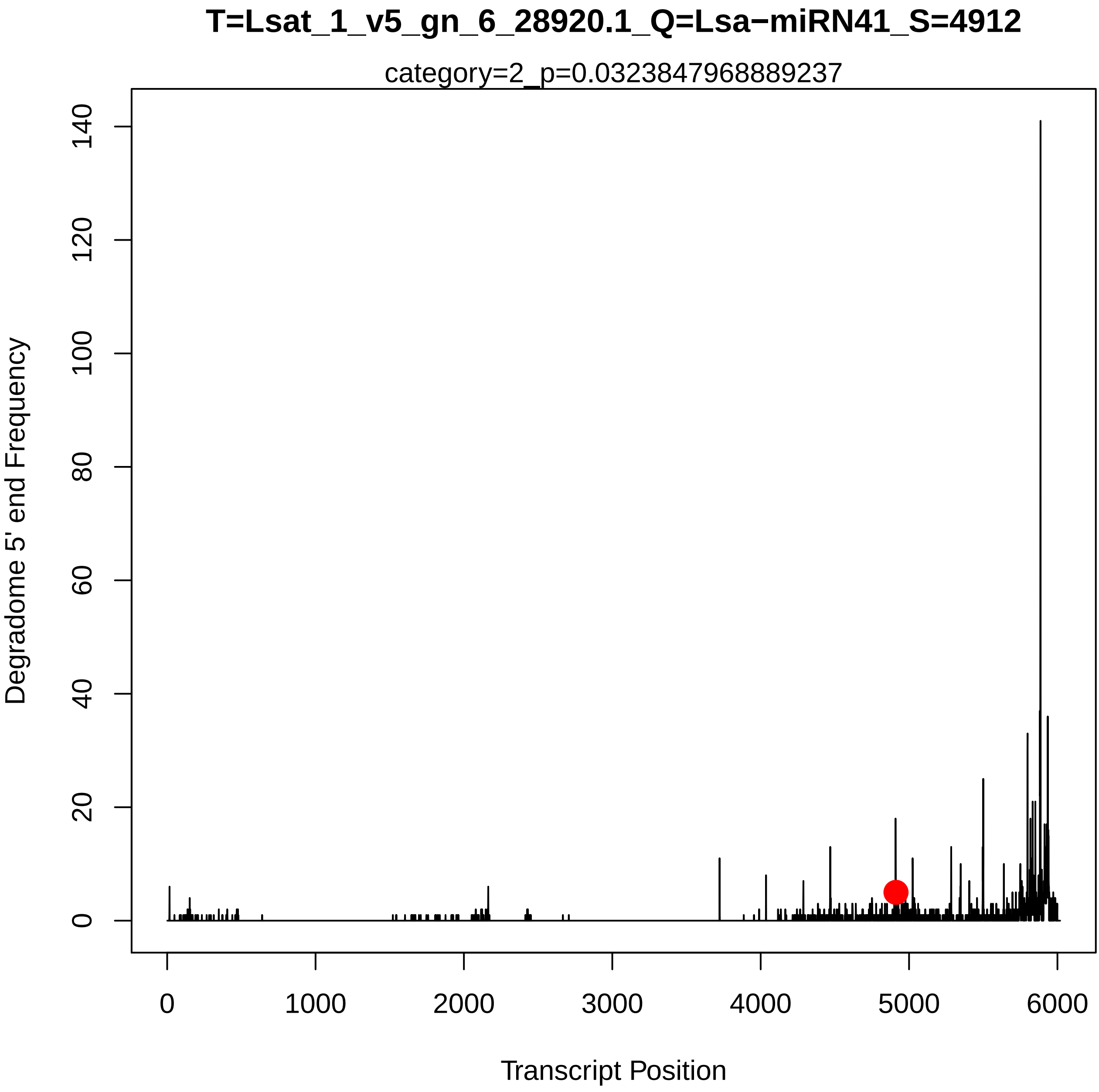
T=Lsat_1_v5_gn_6_28920.1_Q=Lsa−miRN41_S=4912
category=2_p=0.0323847968889237
Degradome 5' end Frequency
0
20
40
60
80
100
120
140
0
1000
2000
3000
4000
5000
6000
Transcript Position
